# Increased urbanization reduced the effectiveness of school closures on seasonal influenza epidemics in China

**DOI:** 10.1186/s40249-021-00911-7

**Published:** 2021-10-21

**Authors:** Hao Lei, Hangjin Jiang, Nan Zhang, Xiaoli Duan, Tao Chen, Lei Yang, Dayan Wang, Yuelong Shu

**Affiliations:** 1 School of Public Health, Zhejiang University, Hangzhou, People’s Republic of China; 2 Center for Data Science, Zhejiang University, Hangzhou, People’s Republic of China; 3 Key Laboratory of Green Built Environment and Energy Efficient Technology, Beijing University of Technology, Beijing, People’s Republic of China; 4 School of Energy and Environmental Engineering, University of Science and Technology Beijing, Beijing, China; 5 National Institute for Viral Disease Control and Prevention, Collaboration Innovation Center for Diagnosis and Treatment of Infectious Diseases, Chinese Center for Disease Control and Prevention; Key Laboratory for Medical Virology, National Health Commission, Beijing, 102206 People’s Republic of China; 6 School of Public Health (Shenzhen), Shenzhen Campus of Sun Yat-Sen University, No. 66, Gongchang Road, Guangming District, Shenzhen, Guangdong 518107 People’s Republic of China

**Keywords:** School closure, Influenza epidemics, Urbanization, Contact, China

## Abstract

**Background:**

School closure is a common mitigation strategy during severe influenza epidemics and pandemics. However, the effectiveness of this strategy remains controversial. In this study, we aimed to explore the effectiveness of school closure on seasonal influenza epidemics in provincial-level administrative divisions (PLADs) with varying urbanization rates in China.

**Methods:**

This study analyzed influenza surveillance data between 2010 and 2019 provided by the Chinese National Influenza Center. Taking into consideration the climate, this study included a region with 3 adjacent PLADs in Northern China and another region with 4 adjacent PLADs in Southern China. The effect of school closure on influenza transmission was evaluated by the reduction of the effective reproductive number of seasonal influenza during school winter breaks compared with that before school winter breaks. An age-structured Susceptible-Infected-Recovered-Susceptible (SIRS) model was built to model influenza transmission in different levels of urbanization. Parameters were determined using the surveillance data via robust Bayesian method.

**Results:**

Between 2010 and 2019, in the less urbanized provinces: Hebei, Zhejiang, Jiangsu and Anhui, during school winter breaks, the effective reproductive number of seasonal influenza epidemics reduced 14.6% [95% confidential interval (*CI*): 6.2–22.9%], 9.6% (95% *CI:* 2.5–16.6%), 7.3% (95% *CI:* 0.1–14.4%) and 8.2% (95% *CI:* 1.1–15.3%) respectively. However, in the highly urbanized cities: Beijing, Tianjin and Shanghai, it reduced only 5.2% (95% *CI:* -0.7–11.2%), 4.1% (95% *CI:* -0.9–9.1%) and 3.9% (95% *CI:* -1.6–9.4%) respectively. In China, urbanization is associated with decreased proportion of children and increased social contact. According to the SIRS model, both factors could reduce the impact of school closure on seasonal influenza epidemics, and the proportion of children in the population is thought to be the dominant influencing factor.

**Conclusions:**

Effectiveness of school closure on the epidemics varies with the age structure in the population and social contact patterns. School closure should be recommended in the low urbanized regions in China in the influenza seasons.

**Graphical abstract:**

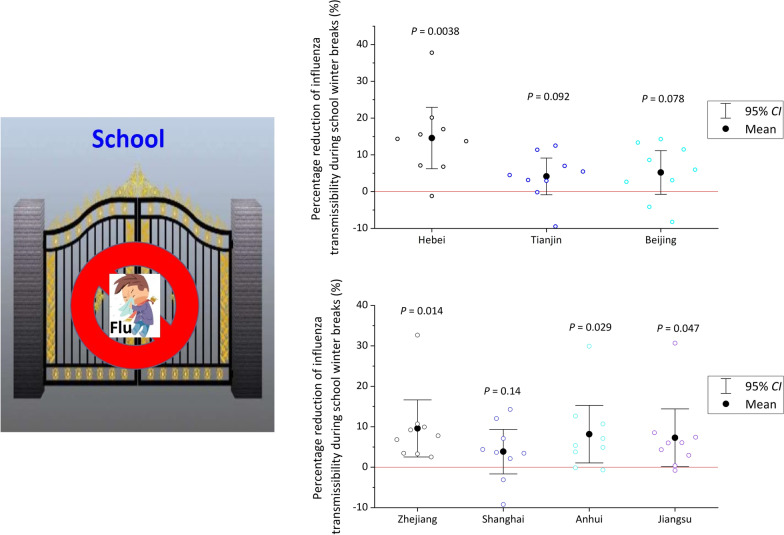

**Supplementary Information:**

The online version contains supplementary material available at 10.1186/s40249-021-00911-7.

## Background

School-aged children are a key source of influenza transmission. This is largely because they have higher social contact [1] and are more susceptible to seasonal influenza virus than adults [2]. School closure is a common mitigation strategy to generate social distancing during severe influenza epidemics and pandemics [3]. However, debates over the effect of school closure on influenza transmission are rife [4]. Studies in France [5] and Republic of Korea [6] report scheduled school breaks to be associated with moderate reductions in influenza transmission. However, a study in Hong Kong, China showed that school closure did not have a significant effect on influenza transmission [7]. In China, seasonal influenza epidemics peak in January–February and the duration is approximately 11–14 weeks [8]. School winter breaks begin in January and last for around one month. The overlap between school winter breaks and the winter-spring waves of influenza epidemics provides a unique opportunity to explore the impact of school closure on seasonal influenza epidemics.

The level of influenza transmission occurring in schools contributed to the impact of school closure on influenza epidemics [4]. For example, if 50% of transmission occurs in schools, it is likely that school closure would be highly effective in reducing influenza spread. Whereas, if only 10% of transmission occurs in schools, a much weaker effect could be expected. The increased proportion of school-aged children in the population could be associated with increased transmission rates of influenza. In China, increased urbanization is associated with increased population age. Therefore, the proportion of school-aged people decreases significantly with increased urbanization. We assume that urbanization level in China is an important factor that contributes to effectiveness of school closure on seasonal influenza epidemics. In China, urbanization varies significantly between provincial-level administrative divisions (PLDS). For example, urbanization rates of PLADs in China ranged from 47.5% to 88.1% in 2018 [9]. This variation together with the aforementioned overlap between school closure and influenza epidemic waves in China presents a unique opportunity to study the role of urbanization on the effectiveness of school closure on influenza epidemics within a single health care system.

In this study, influenza surveillance data from 2010 to 2019 in China was analyzed to explore the impact of winter school breaks on seasonal influenza transmission in PLADs with varying urbanization rates.

## Methods

### PLADs involved in this study

To control the random effect, PLADs in China with different urbanization rates were selected for analysis. In 2018, Beijing, Tianjin and Shanghai have the highest urbanization rates and the rates were 86.5%, 83.2% and 88.1% respectively [9]. Note that these PLADs have different climate, and it is reported that climate influences the spread of influenza [10, 11]. To control the effect of climate on the analysis, PLADs around Beijing, Tianjin and Shanghai were selected. Taking into account climate and the urbanization level, we defined two regions. Region 1 locating in Northern China includes three PLADs: Beijing, Tianjin and Hebei. Geographically, Tianjin and Beijing are surrounded by Hebei. In 2018, the urbanization rate in Hebei was 56.4%, which is much lower than that in Beijing (86.5%) and Tianjin (83.2%) [9]. Region 2 locating in Southern China includes Shanghai, Zhejiang, Jiangsu and Anhui. Shanghai is surrounded by Zhejiang, Jiangsu and Anhui. The urbanization rates in Zhejiang, Jiangsu and Anhui in 2018 were 68.9% 69.6% and 54.7% respectively [9]. These rates are lower compared with Shanghai (88.1%).

### Data sources

In China, the influenza epidemiological year is from 1 October to 30 September of the next year [8]. Weekly reports of influenza surveillance data from these selected seven PLADs between 1 October 2010 and 30 September 2019 were downloaded from WHO Flunet (www.who.int/influenza/gisrs_laboratory/flunet/en/). This data was originally provided by the Chinese National Influenza Center. The weekly reports include the number of hospital visits, cases of influenza-like illness (ILI), specimens tested, as well as the number of laboratory-confirmed cases of influenza A (H1N1, H3N2, and pdmH1) and B (Yamagata and Victoria). Similar to previous studies [12, 13], a proxy for the weekly incidence rate (referred to as the ‘incidence rate’) was estimated, which was calculated by multiplying the ILI rate among patients visiting sentinel hospitals by the viral detection positive rate. Using the weekly incidence rate, this proxy precisely represents the incidence of influenza infection. The daily incidence rate was calculated using splines [14].

### Models

At the peak of an influenza outbreak, the incidence rates will reduce even if there is no intervention. Therefore, comparing incidence rates before and during school closure could potentially be misleading [4]. In this study, the impact of school closure on seasonal influenza epidemics was measured by the changes of the effective reproductive number of influenza epidemics, *R*_*t*_, between before and during school closure. *R*_*t*_ is the mean number of secondary infections caused by a primary infection at time *t* [6, 7], which was estimated using the *R* package EpiEstim with a mean serial interval of 2.85 days and a standard deviation of 0.93 days [15]. The difference on mean of *R*_*t*_ during the school winter breaks and during the two weeks before the school winter breaks is used to evaluate the impact of school closure on influenza transmission [6]. School winter breaks in China always begin on the Lunar calendar 15 December and end on the Lunar calendar 15 January, and may have a small variation. This study defined school winter breaks to be 15 days before and after Chinese New Year's Eve. This definition is critical to the study, as the timing of school closure is an important factor to determine its impact [16]. For example, if school closure occurs when the effective reproductive number is already below one, its impact on transmission would be ignorable. In this study, these nine epidemiological years were divided into two groups. The first group includes years when school closure occurred early, i.e. the start of school closure occurred before the peak time of influenza epidemic. The second group includes years when school closure occurred late, i.e. school closure begun after the influenza epidemic peak time.

We proposed an age-structured Susceptible-Infected-Recovered-Susceptible (SIRS) model to explore the association between urbanization level and the effect of school closure on seasonal influenza epidemics in China. In this model, we considered three age groups (1) children aged < 14, (2) people aged 14–65, and (3) elderly aged > 65. This categorization was based on age-specific susceptibility to influenza virus [17]. The governing equations are as follows:

$$\frac{{dS_{i} \left( t \right)}}{dt} = \frac{{N_{i} - S_{i} \left( t \right) - I_{i} \left( t \right)}}{L} - \frac{{\mathop \sum \nolimits_{j = 1}^{3} \beta_{ij} \left( t \right)S_{i} \left( t \right)\left( {\mathop \sum \nolimits_{\tau = 1}^{7} P_{\tau } I_{j} \left( {t - \tau } \right)} \right)}}{{N_{i} }}$$

$$\frac{{dI_{i} \left( t \right)}}{dt} = \frac{{\mathop \sum \nolimits_{j = 1}^{3} \beta_{ij} \left( t \right)S_{i} \left( t \right)\left( {\mathop \sum \nolimits_{\tau = 1}^{7} P_{\tau } I_{j} \left( {t - \tau } \right)} \right)}}{{N_{i} }} - \frac{{I_{i} \left( t \right)}}{D}$$

$$\frac{{dR_{i} \left( t \right)}}{dt} = \frac{{I_{i} \left( t \right)}}{D} - \frac{{N_{i} - S_{i} \left( t \right) - I_{i} \left( t \right)}}{L}$$

where $P_{\tau }$ is the 7 day infectivity profile chosen as a gamma distribution with a mean of 2.7 days and variance of 1.8 days [18]. Then the number of new infections at time *t* is defined as:

$$i\left( t \right) = \frac{{\mathop \sum \nolimits_{i}^{3} \mathop \sum \nolimits_{j = 1}^{3} \beta_{ij} \left( t \right)S_{i} \left( t \right)\left( {\mathop \sum \nolimits_{\tau = 1}^{7} P_{\tau } I_{j} \left( {t - \tau } \right)} \right)}}{{N_{i} }}$$

$S_{i} \left( t \right)$, $I_{i} \left( t \right)$ and $R_{i} \left( t \right)$ are the susceptible, infected and recovered population in the $i$ age group, respectively. *i* = 1, 2, 3 corresponds to the children aged < 14, individual aged 14–65, and the elderly aged > 65 respectively. $N_{i}$ is the total number of population in the $i$-th age group. The total population number is $N = \mathop \sum \limits_{i = 1}^{3} N_{i}$. $\beta_{ij} \left( t \right) = \beta \left( t \right)c_{ij} \gamma_{i}$, where $\beta \left( t \right)$ is the transmission efficiency per contact at time *t*. The impact of climate on influenza transmission, was modelled by the equation: $\beta \left( t \right) = a$(1 $+ b$ sin(*wt* + *c*)) [19], where *b* represents the relative contribution of climates on influenza transmission efficiency, and typically takes values in the range (0.05, 0.3) [20]. $\gamma_{i}$ represents the susceptibility of age $i$-th group to the virus, in this study, we take $\gamma_{1}$:${ }\gamma_{2}$:${ }\gamma_{3}$ = 2:1:2 [5]. $L$ and $D$ denote the immunity and infectious period respectively. Following the previous work, we take $L$ = 730 days and $D$ = 3 days [15, 21]. Sensitivity analysis of $L$ and $D$ are given in Additional file 1: S3. $c_{ij}$ denotes the contact rates between the $i$ and *j* group. Contact rates in Wuhan and Shanghai were obtained from Zhang et al. [22]. Contact rates in PLADs with low urbanization levels (Hebei, Zhejiang, Anhui and Jiangsu) were estimated using survey data in Wuhan. Contact rates in Beijing, Tianjin and Shanghai were estimated using survey data in Shanghai. During school winter breaks, it was assumed that contact rates between young people reduced to 1.5, since during school closure, the mean total number of contacts for each student would reduce 65% [23]. Other contact rates were unchanged. Detailed contact rates $c_{ij}$ are described in the Additional file 1: S1.

In the SIRS model, the effective reproductive number is estimated as:

$$R_{t} = \frac{{\mathop \sum \nolimits_{i = 1}^{3} \left( {\mathop \sum \nolimits_{j = 1}^{3} \beta \left( t \right)c_{ij} \gamma_{i} N_{i} } \right)}}{N}D$$

Finally, unknown parameters, $a$, *b*, *c*, and *w,* in the model, are estimated by Bayesian method, details are given in Additional file 1: S2. In the simulation, the initial condition of I, R and S were ${\uptheta }$ N, 0 and N-${\uptheta }$ N, where ${\uptheta }$ is the 40% quantile of the non-zero weekly incidence rate, which has been used in literatures to identify the onset of an influenza season [24, 25].

In the highly urbanized PLADs (Beijing, Tianjin and Shanghai), the proportion of children is relatively lower and contact rates between individuals are higher. Both factors may contribute to the effectiveness of school closure on seasonal influenza epidemics. The effect of the proportion of children on school closure during seasonal influenza epidemics is well understood. If social contact is close within a population, it is likely that reducing the contact among children by school closure would have a lower impact on influenza transmission rates. Therefore, this study analyzed four key scenarios to quantify the relative importance of these factors:

*Scenario 1* High contact rates between individuals and a low proportion of children (in Beijing and Tianjin).

*Scenario 2* Low contact rates between individuals and a high proportion of children (in Hebei).

*Scenario 3* High contact rates between individuals and a high proportion of children.

*Scenario 4* Low contact rates between individuals and a low proportion of children.

For each of these, the effect of school closure on seasonal influenza epidemics was calculated. Hebei was considered to have a high proportion of children (18.5%) [9] and Beijing (Tianjin) was determined to have a low proportion of children (10.3%) [9]. High and low contact rates were determined by the contact rates in Beijing and Hebei respectively, which have relatively high and low level of urbanization, respectively.

Data was analyzed using the software R 3.63 (The R Project for Statistical Computing, Guangzhou, China). Statistical significance was determined using the *t*-tests, and two-sided *P* values are reported.

## Results

The PLADs within each region are neighbours to each other. Therefore, the temperature and relative humidity of the 3 PLADs within region 1 and the 4 within region 2 are similar (Table 1). In addition, the relative contribution of climate on influenza transmission efficiency was typically less than 30% [20]. This suggests that within each region, the influence of climate on the analysis is under control.

**Table 1:** Background characteristics of the seven provincial-level administrative divisions in region 1 and region 2 during 2010–2019

	Region 1			Region 2			
	Hebei	Tianjin	Beijing	Shanghai	Zhejiang	Jiangsu	Anhui
Cities (hospitals)^a^	10 (24)	1 (10)	1 (11)	1 (19)	12 (16)	13 (29)	17 (25)
Population size (million)^b^	74.25	15.47	21.71	24.15	55.39	79.76	55.39
Latitude (°N)	38.1	39.2	39.9	31.3	30	32.9	31.8
Longitude (°E)	115.8	117.2	116.4	121.5	120.4	118.6	117.5
Mean winter temperature (°C)	1.2	3.5	1.8	10.5	11.3	8.2	8.6
Mean winter relative humidity (%)	54.6	54.4	49.9	69.5	74.1	71.8	70.4
Urbanization rate^c^	49.3%	82.3%	86.5%	87.6%	67.0%	67.7%	50.5%

^a^Number of cities and hospitals in the surveillance

^b^Population size in millions in 2015 [9]

^c^Urbanization rate in 2015 [9]

### Effects of school closure on epidemics by PLAD

**Fig. 1:**
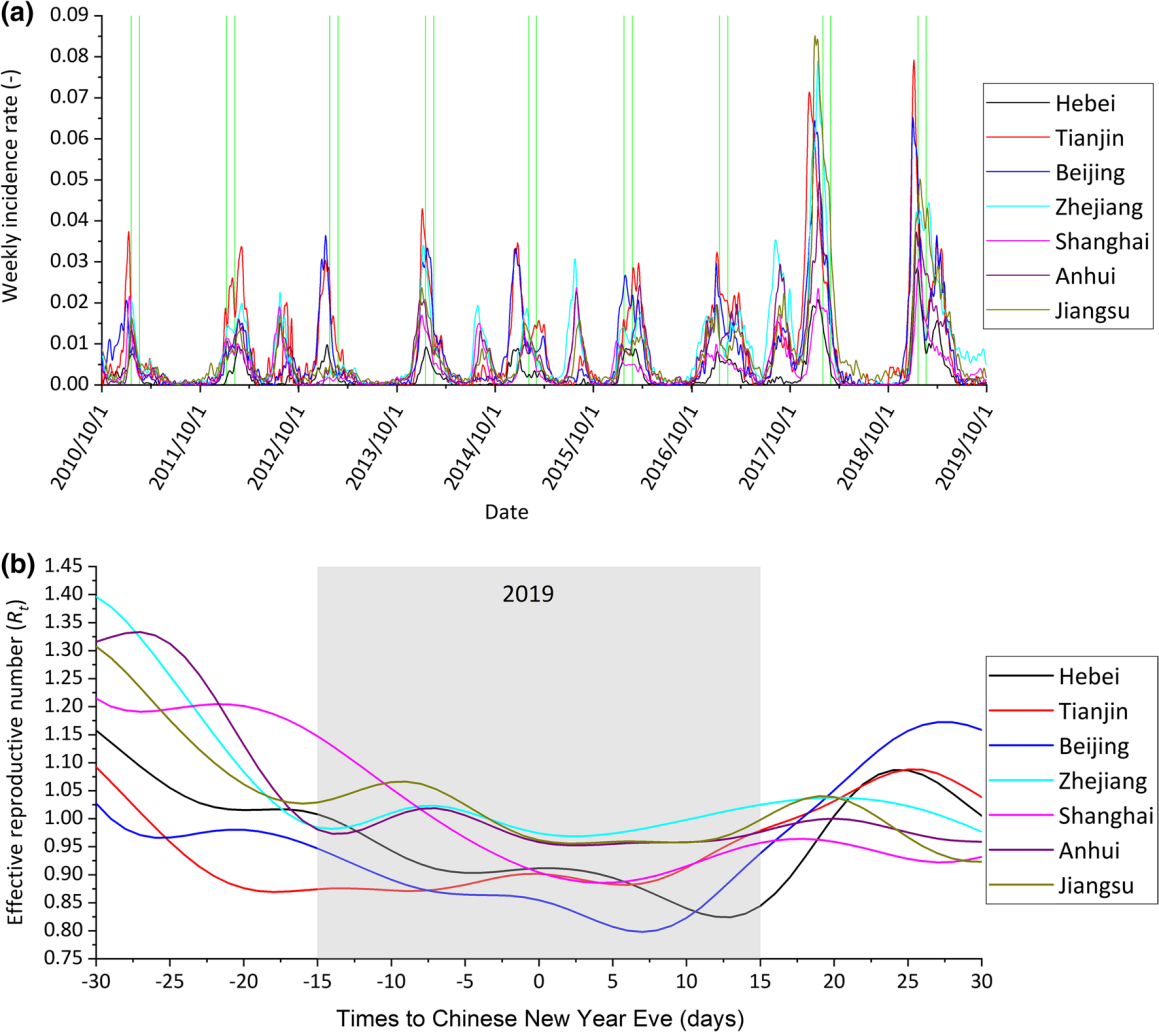
**a** Weekly influenza incidence rates from 1 October 2010 to 30 September 2019. The two adjacent vertical green lines denote the timing of the school winter breaks; **b** Daily reproductive number of influenza before, during and after school winter breaks in 2019. The gray area denotes the winter school break period

**Fig. 2:**
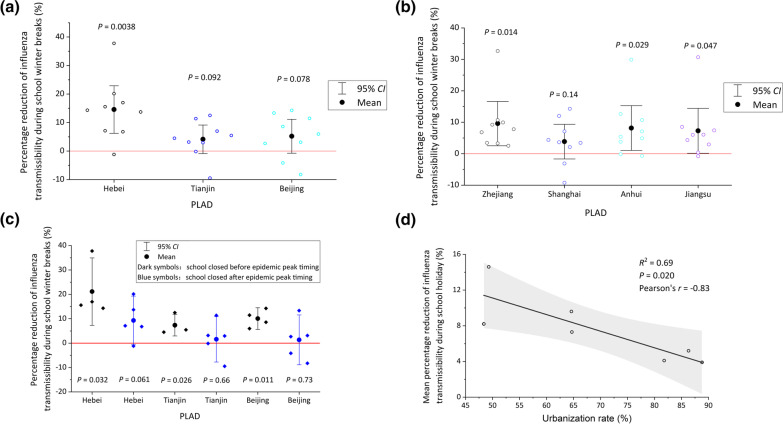
Reduction in influenza transmissibility during school winter breaks, **a** in Hebei, Tianjin and Beijing within region 1; **b** in Zhejiang, Shanghai, Anhui and Jiangsu within region 2 in the year 2010–2019. *P* value indicated statistically significant reductions in influenza transmissibility; **c** in different years when school closure started before or after the epidemic peak. Black symbols denote percentage reduction in 2012, 2014, 2016, and 2019, when school closure started before the epidemic peak. Blue symbols denote when school closure stared after the peak, in 2011, 2013, 2015, 2017, and 2018 in Hebei, Tianjin and Beijing; **d** Mean reduction with urbanization rates. The gray area shows the 95% *CI* of the linear fit. PLAD: Provincial-level administrative division, *CI*: Confidential interval

In China, school winter breaks overlap with the winter-spring waves of influenza epidemics (Fig. 1a). As an example, in 2019, when the school winter breaks started, the effective reproductive number of seasonal influenza decreased. After the school winter breaks, the number increased again (Fig. 1b). In Hebei, the mean reduction in influenza transmissibility during the winter school breaks was 14.6% [95% confidential interval (*CI*): 6.2–22.9%]. In Tianjin this reduction was much lower (4.1%, 95% *CI:* -0.9–9.1%) (*P* = 0.002). A low reduction was also observed in Beijing (5.2%, 95% *CI:* -0.7–11.2%) (*P* = 0.02) (Fig. 2a). An exception to this trend occurred in 2015. In this epidemiological year, there was no decrease in influenza transmissibility observed during winter school breaks in all 3 PLADs of region 1. For 4 PLADs in region 2, the mean percentage reduction of influenza transmissibility in Shanghai was 3.9% (95% *CI:* -1.6–9.4%) during winter school breaks. The reduction of influenza transmissibility observed was more sizeable in Zhejiang (9.6%, 95% *CI:* 2.5–16.6%), Jiangsu (7.3%, 95% *CI:* 0.1–14.4%) and Anhui (8.2%, 95% *CI:* 1.1–15.3%) (Fig. 2b). In summary, in the highly urbanized PLADs, Beijing, Tianjin and Shanghai, influenza transmissibility did not decrease significantly during the school winter breaks. However, in the less urbanized PLADs, Hebei, Zhejiang, Jiangsu and Anhui, a significant decrease in influenza transmissibility was observed (Fig. 2a, b).

The effectiveness of school closure on influenza epidemics varied with the timing of the school closure (Fig. 2c). When school closure occurred late in the epidemic, it was less effective. Even in the less urbanized PLAD, Hebei, the reduction of effective reproductive number during school closure was not statistically significant (*P* = 0.061) (Fig. 2c). However, it is important to note that whether school closure occurred early or late in the epidemic, the reduction in influenza transmissibility during the winter school breaks was higher in the low urbanized Hebei than that in the high urbanized Beijing and Tianjin (Fig. 2c). There was a statistically significant decrease in the impact of school closure on influenza transmission with increased urbanization in the 7 PLADs (Fig. 2d).

### Simulated relative role of contributing factors

**Fig. 3:**
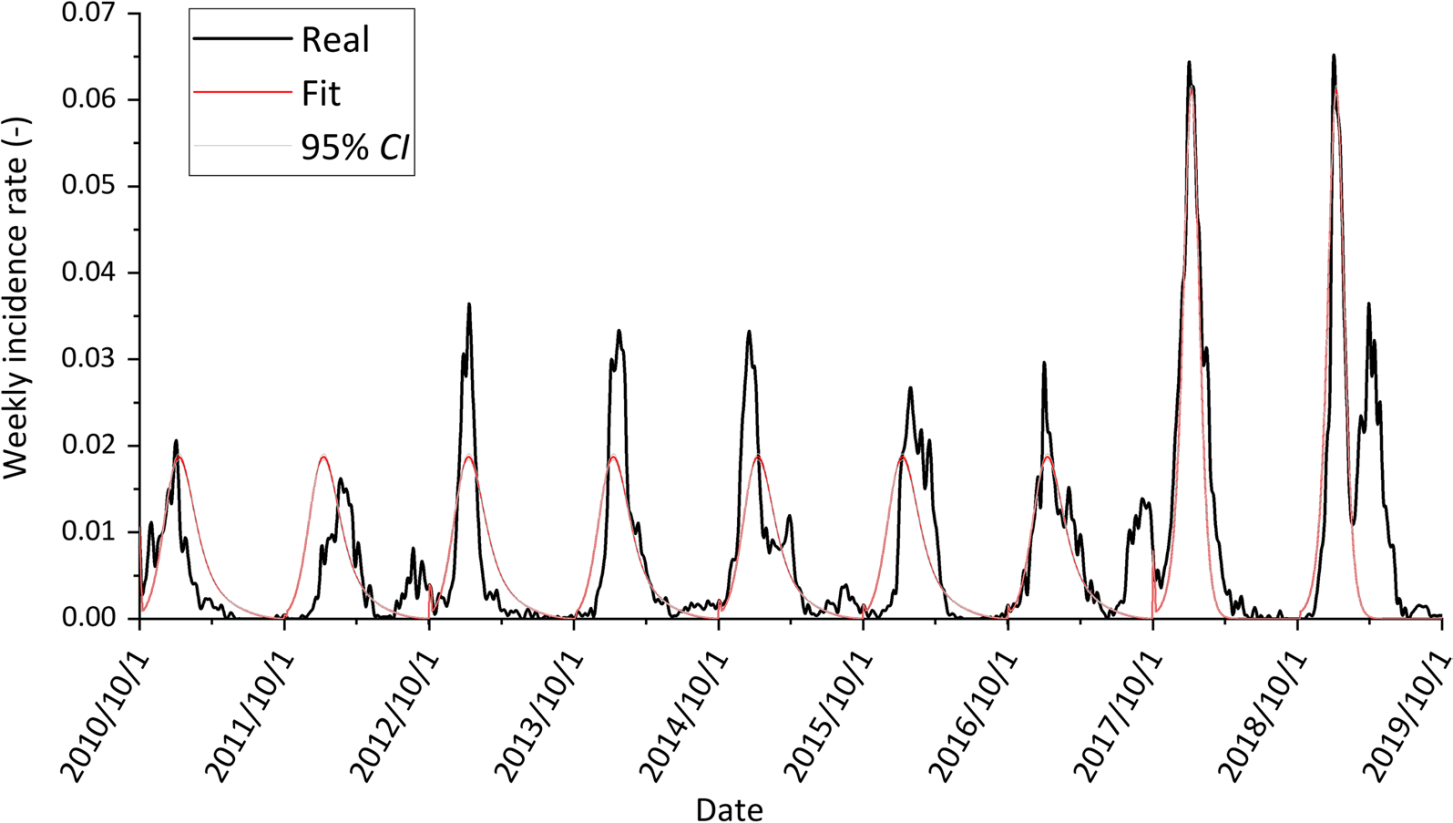
Influenza weekly incidence rates in Beijing from 1 October 2010 to 30 September 2019. Red denotes fitting model outcome and gray denotes 95% *CI*. *CI*: Confidential interval

**Fig. 4:**
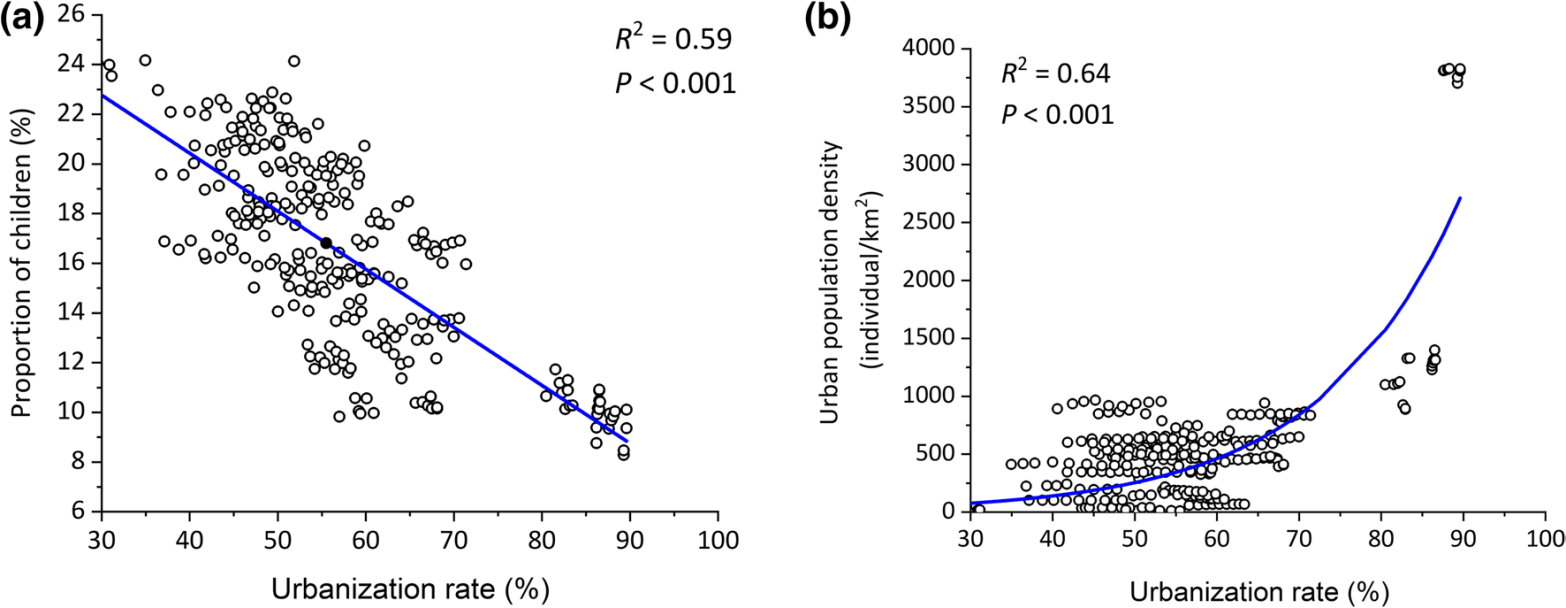
Population distribution in provincial-level administrative divisions with differing urbanization in China from 2010 to 2019. **a** Proportion of children < 14 years; **b** urban population density

Our simulation was divided into two periods. The first was from 1 October 2010 to 30 September 2017, when influenza prevalence was relatively low. The second was from 1 October 2017 to 30 September 2019, when influenza prevalence was relatively high (Fig. 3). Detailed parameter inference is described in Additional file 1: S2. The proportion of children younger than 14 decreased significantly from 2010 to 2019 with increased urbanization in China (Fig. 4a). It is expected that the urban population density and thus the social contact increase with the urbanization across China (Fig. 4b). Based on the estimated parameters obtained from surveillance data of Beijing, the reduction of influenza transmissibility during school winter breaks was estimated in the 4 scenarios. As expected, when the influenza prevalence was high, school closure is more effective on the epidemic (Table 2). When the population have the same level of social contact, and the proportion of children decreased from 18.4% to 10.4%, there was a 5.0–5.3% reduction in influenza transmissibility during school winter breaks (scenario 1 vs scenario 3). There was a 5.4–5.6% reduction for scenario 2 vs scenario 4 (Table 2). In scenarios with the same proportion of children and increased contact rates, the percentage reduction in influenza transmissibility during school winter breaks was only 1.6% (scenario 3 vs scenario 2) and 1.2–1.3% (scenario 4 vs scenario 1).

**Table 2:** Estimated reductions in influenza transmissibility during school winter breaks in four different scenarios via the SIRS model

	Percentage reductions in influenza transmissibility during winter school breaks	
	Low prevalence (95% *CI*)	High prevalence (95% *CI*)
Scenario 1	5.0% (1.9–7.8%)	8.0% (7.2–9.1%)
Scenario 2	11.9% (9.0–14.5%)	14.6% (13.9–15.7%)
Scenario 3	10.3% (7.3–12.9%)	13.0% (12.3–14.1%)
Scenario 4	6.3% (3.2–9.0%)	9.2% (8.4–10.3%)

SIRS, Susceptible-Infected-Recovered-Susceptible; *CI*, Confidential interval

## Discussion

In this study, the impact of school closure on seasonal influenza epidemics was investigated in 7 PLADs of China with varying urbanization rates. The key finding was that with increased urbanization, the effectiveness of school closure on seasonal influenza epidemics decreased significantly. This is potentially due to the fact that with increased urbanization in China, the proportion of children aged < 14 decreased significantly. It is likely that a lower proportion of school-aged children corresponds to a lower proportion of influenza cases transmitted in schools. In addition, with increased urbanization, the social contacts also increased, thus children played a less important role in seasonal influenza epidemics.

Consistent with our findings, a study in the Republic of Korea reported scheduled spring school breaks to be associated with 6–23% reduction in *R*_*t*_ of seasonal influenza from 2014 to 2016 [6]. Similarly, a study in France showed that school holidays prevent 16–18% of seasonal influenza cases [5]. It’s important to note that Cauchemez et al. [5] did not estimate the changes in *R*_*t*_. However, it could be expected that reduction in *R*_*t*_ is less than 16–18%. In Israel, winter holidays were associated with reduction of influenza transmission. However, this was only shown in 3 of the 5 seasonal influenza study periods [26]. Contrastingly, a study in Hong Kong, China reported that school closure did not result in *R*_*t*_ reductions in 2008 winter influenza season [7]. Interestingly, Hong Kong is completely urbanized and the proportion of children less than 14 years old is only 12.7% [27]. The urbanization rate in France and the Republic of Korea is approximately 81%, which is lower than that in Hong Kong, China and Israel (100% and 93%, respectively) [27, 28]. These differences in urbanization may explain the varying effects of school closure on seasonal influenza epidemics.

When applying the findings in this study in practice, one should note that not only urbanization, other factors could also potentially contribute to the effect of school closure on influenza epidemic. In this study, we showed timing of school closure to be a key influential factor. Thus, one potential reason for no decrease of the effective reproductive of influenza epidemics in 2015 is that the school winter breaks started when the influenza epidemic had almost ended (Fig. 1a). Modelling studies suggested that if school closure occurs before 1% of the population get sick, the effect is maximal [29]. In this study, in the less urbanized Hebei, if school closure occurred after the peak of the epidemic, there was no statistically significantly decrease in the effective reproductive number of influenza (Fig. 2c). This timing may be the underlying factor that explains why some studies reported no clear benefit of school closure [7, 30]. Although the effect of school closure in PLADs with similar climate were compared to control the impact of climate on the analysis, their climates were still more or less different. For example, in the less urbanized Anhui, the effect of school closure are similar to that in the higher urbanized Zhejiang and Jiangsu, the main reason could be that the climate of Anhui is drier than that of Zhejiang and Jiangsu (Table 1), and influenza virus favors low humidity [10], so the influenza epidemic in Anhui sometime would start a little earlier, with same timing of school closure, school closure in Anhui would be less effective. This study showed that compared with higher urbanized PLADs, reductions in influenza transmissibility observed during winter school breaks were higher in less urbanized PLADs (Fig. 2c). In other words, increased urbanization in China reduced the effectiveness of school closure on seasonal influenza epidemics.

The findings from seasonal influenza epidemic data may not be directly applicable to a pandemic [16]. Importantly, although the benefits of school closure are predicted to be substantial, it is not always recommended in seasonal influenza epidemics, since we should take the potential high economic and social costs of school closure into account. However, during influenza pandemics, this strategy is always recommended. As a result, there are a plethora of studies investigating the effect of school closure on influenza pandemics. Previous studies on influenza pandemics have suggested that sustained school closure during a pandemic could reduce peak attack rates and prevent 13–17% of cases in France [5] and < 20% of cases in the United Kingdom [31]. One study in Hong Kong showed that school closure during the 2009 H1N1 pandemic reduced the effective reproductive number by 11.8% [32]. In Mexico and New Zealand, there also have been reported decreases in *R*_*t*_ during school closure [33, 34]. Contrastingly, in the 2009 pandemic in USA there was no clear effect of school closure detected on transmission [30]. This was mainly due to the late timing of the closures. During the 1957 pandemic in France, school closure were judged to be ineffective, which is mainly due to that 50–75% of the population had been ill when schools were closed [4]. During the 1918 influenza pandemic, school closure was performed in combination with other interventions. As a result, it is impossible to determine its specific effect [4]. Aside from the study in the USA during the 2009 pandemic, where school closure was ineffective because schools were closed late, other studies have shown that school closure is effective against influenza pandemics. This study showed that school closure was most effective when the epidemics were at relatively high prevalence level (Table 2). This offers a potential explanation on why school closure was ineffective during an epidemic but effective during the 2009 pandemic in Hong Kong, China [7, 32].

This study has various limitations. First, we found that the decreasing effect of school closure on epidemics with increasing urbanization was mainly linked to decreasing numbers of school-aged children. However, the level of urbanization potentially also influences influenza transmission in other ways. For example, highly urbanized areas for the most part have access to superior medical services, which could facilitate more rapid treatment of influenza-infected children, and then as such, better control of influenza transmission within schools. However, approximately 90% of seasonal influenza cases are unreported [35]. Infectious cases are most transmissible during the incubation period and the first 2–3 days with clinic symptoms. Therefore, we predict that quality of medical services would have a limited impact on influenza epidemics. Second, our model did not consider differences in influenza vaccine coverage because this rate has increased only gradually to approximately 2% of the Chinese population over the past 15 years [36]. This low influenza vaccination rate would have a very limited influence on an epidemic. Third, the contact rates leveraged in the modelling study. Due to the lack of data, it was assumed that during school winter breaks, contact between students decreased, but other contact rates remain unchanged. However, the Lunar New Year holidays is within the school winter breaks. People have enhanced social contact during this time. This limitation could be addressed if more contact data was available for before and after school closure. The last limitation of this study is the interpolation of daily incidence rates of influenza from weekly data. The daily variation in influenza transmissibility may have been reduced. The availability of daily data of influenza would address this limitation.

## Conclusions

Effectiveness of school closure on the seasonal influenza epidemics could be influenced by the population age structures and social contact patterns. In the less urbanized regions with relative higher proportion of children in the population and less contact between adults, school closure is associated with the reduction of effective reproductive number of influenza epidemics, thus school closure should be recommended in the low urbanized regions in China during severe influenza epidemic or pandemic.

## Supplementary Information

**Additional file 1.** Parameter inference and sensitivity analysis.

## Data Availability

The datasets used in the study are available from the corresponding author on reasonable request.

## Abbreviations

PLADs: Provincial-level administrative divisions
ILI: Influenza-like illness
*CI*: Confidential interval
SIRS: Susceptible-Infected-Recovered-Susceptible
