# Supplementary material for: Increased urbanization reduced the effectiveness of school closures on seasonal influenza epidemics in China
Source: Infect Dis Poverty. 2021 Oct 21;10:127. doi: 10.1186/s40249-021-00911-7 (PMC8532386; doi:10.1186/s40249-021-00911-7)
Supplement: Supplementary file 1 — Additional file 1. Parameter inference and sensitivity analysis. [file 40249_2021_911_MOESM1_ESM.docx]

**Supplements**

**Increased urbanization reduced the effectiveness of school closures on seasonal influenza epidemics in China**

Hao Lei^1#^, Hangjin Jiang^2#^, Nan Zhang^3^, Xiaoli Duan^4^, Tao Chen^5^, Lei Yang^5^, Dayan Wang^5^, Yuelong Shu^6*^

**1. Contacts between different age groups**

The contact levels presented by Zhang et al., 2020 were used to estimate the contact rates between different age groups. The contact rate, $C_{LU},$ between the 3 age groups before school winter breaks in Hebei, Zhejiang, Anhui and Jiangsu were estimated from survey data from Wuhan. The contact rates, $C_{HU},$ in Beijing and Tianjin and Shanghai were estimated using the data from Shanghai. In the study performed by Zhang et al., 2020, the mean contact rate between young people in Wuhan was estimated to be 1.67 per day. This value seems fairly low. The mean contact rate between young people in Shanghai was estimated to be 4.13 per day. As these young people are in school, the contact rates between students shouldn’t vary so vastly. In this study, the mean contact rate between young people in Hebei was taken to be 3.67 per day. This value is just under the value for Shanghai. Consequently, the contact rate matrix $C_{LU}$ and $C_{HU}$ are as shown below. It’s key to note that as expected, people in highly urbanized regions have higher contact rates than in the less urbanized regions.

$$C_{LU}={{(c}_{ij})}_{3\times3}=\left( \begin{aligned} 3.67 \\ 0.71 \\ 0.68 \end{aligned}\begin{aligned} 0.8 \\ 1.09 \\ 1.16 \end{aligned}\begin{aligned} 0.22 \\ 1.37 \\ 2.91 \end{aligned} \right)_{3\times3}$$

$$C_{HU}={{(c}_{ij})}_{3\times3}=\left( \begin{aligned} 4.13 \\ 0.15 \\ 0.07 \end{aligned}\begin{aligned} 0.51 \\ 1.82 \\ 0.57 \end{aligned}\begin{aligned} 0.72 \\ 1.57 \\ 5.56 \end{aligned} \right)_{3\times3}$$

During school winter breaks, it was assumed that contact rates between young people reduced to 1.5, since during school closure, the mean total number of contacts for each student would reduce 65% (Jackson et al., 2011). Other contact rates were unchanged. This assumption was made based on studies in London and France, which reported that school breaks did not affect contact patterns of adults (Cauchemez et al., 2008; Birrell et al., 2011).

**2. Parameter inference**

In the model, there were 4 unknown parameters. These were $a$, *b*, *c*, and $w,$ and were estimated using the influenza surveillance data. However, these parameters correlated. In particular *a* and *c* correlated highly with *b* and *w*. This makes the model unidentifiable (with many local modes). In order to make the model identifiable, *b* and $w$ were estimated using reasonable values from the literature. Then, a and c were estimated via a robust Bayesian method (Bissiri et al., 2016). In this method, the likelihood function of the observations *Y* is defined as $f\left( Y \right)=exp(-\sum_{t} L\left( i\left( t \right), \hat{i}\left( t \right) \right))$, where $L\left( i\left( t \right), \hat{i}\left( t \right) \right)$ is the loss of using $\hat{i}\left( t \right)$ to estimate the number of new cases at time *t,* $i\left( t \right)$, and *Y* is the total $i\left( t \right)$. As $i\left( t \right)$ is a small value in real observations, the squared loss function is not applicable to capture the deviation between $\hat{i}\left( t \right)$ and $i\left( t \right)$. So, we use the abstract loss function, i.e., $L\left( i\left( t \right), \hat{i}\left( t \right) \right)=k|\hat{i}\left( t \right)-i\left( t \right)|$, where $k$ is a constant to increase the accuracy. In our analysis, we take $k=1000$. Prior distributions for b and c were taken as normal and M-H within Gibbs algorithm is used to sample from posterior distributions.

As influenza occurs annually, as a baseline, $w$ is assumed to be 0.017(=2$\pi$/365), which means $\beta\left( t \right)=a$(1$+b$sin(*wt*+*c*)) is periodic function of 1 year. *b* represented the relative contribution of climate on transmission efficiency, which is typically in the range [0.05, 0.3] (Olinky et al., 2008). For a baseline, *b* = 0.175. In order to evaluate the impact of estimating a and c, sensitivity analysis was performed by taking *b* = 0.125 and 0.225. In the baseline, best fitting parameters [ (mean and 95% confidence intervals (*CI*)] were determined to be a = 0.03780 (95% *CI*: 0.03767, 0.03792); c=-3.10341(95% *CI*: -3.12622, -3.08390). For the first period from 1 October 2010 to 30 September 2017, when the influenza prevalence was relatively low and a = 0.03411(95% *CI*: 0.03399, 0.03421); c = -6.64899 (95% *CI*: -6.67225, -6.62516) during the second period from 1 October 2017 to 30 September 2019, when the influenza prevalence was relatively high.


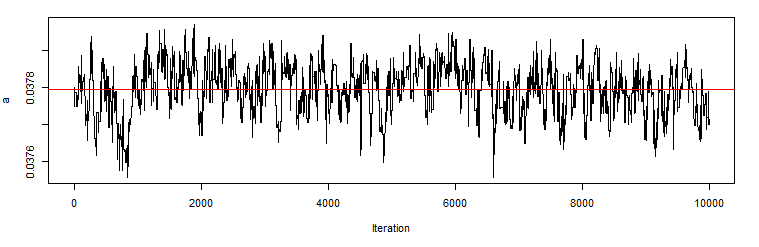


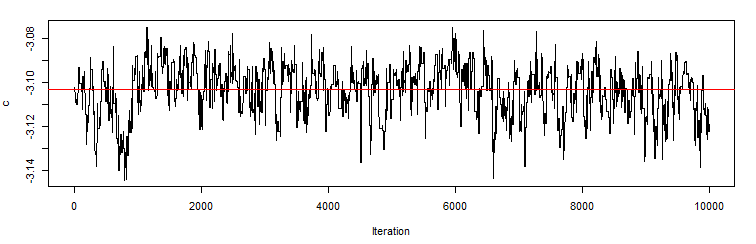


(a)


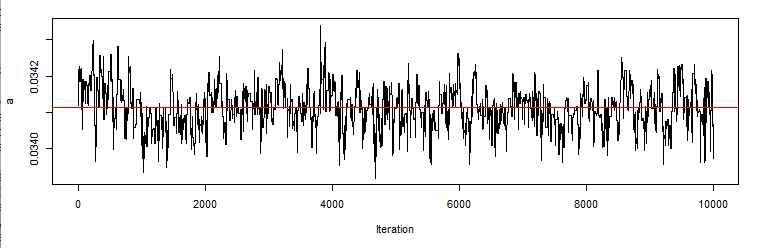


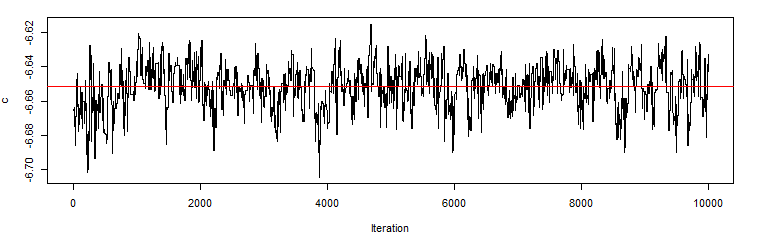


(b)

**Figure S1**. Monte Carlo Markov Chain sampling (after burn-in period) of $a$ and *c* with *b* = 0.175 and $w = 0.017$. (a) from 1 October 2010 to 30 September 2017; (b) from 1 October 2017 to 30 September 2019.

**3. Sensitivity analysis**

**3.1. Sensitivity analysis of parameter *b***

In the baseline, *b* is set to be 0.175. In the sensitivity analysis, *b* is set to be 0.125 and 0.225 respectively. The inference of parameter *a* and *c* was calculated. The percentage reduction in influenza transmissibility during winter school breaks in scenario 1 under low prevalence (95% *CI*) was estimated.

**Table S1**. Inference of parameter $a$ and *c* and percentage reduction in influenza transmissibility during winter school breaks with differing values of parameter *b*.

|  | *b* = 0.125 | *b* = 0.175 (baseline) | *b* = 0.225 |
| --- | --- | --- | --- |
| Parameter $a$ value  (95% *CI*) | 0.03589  (0.03580, 0.03597) | 0.03780  (0.03767, 0.03792) | 0.03988  (0.03976, 0.03998) |
| Parameter *c* value  (95% *CI*) | -3.10832  (-3.13258, -3.08959) | -3.10341  (-3.12622, -3.08390) | -3.12081  (-3.13461, -3.10797) |
| Percentage reduction of influenza transmissibility during winter school breaks in Scenario 1 under low prevalence (95% *CI*) | 6.0%  (3.9%, 7.9%) | 5.0%  (1.9%, 7.8%) | 6.1%  (4.0%, 7.9%) |


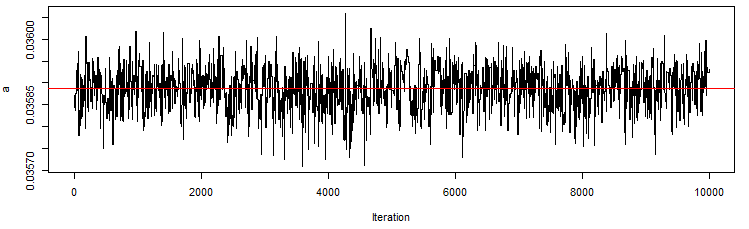


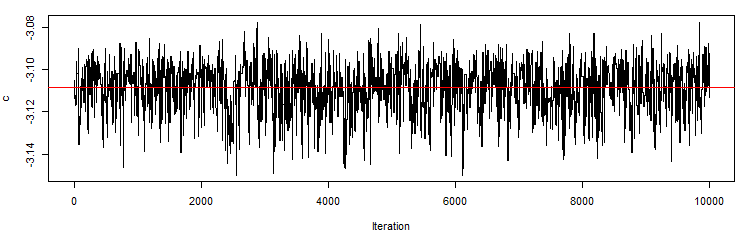


(a)


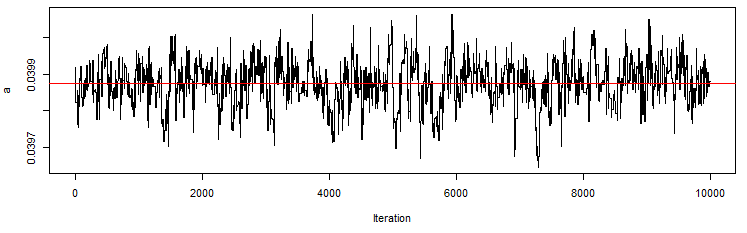


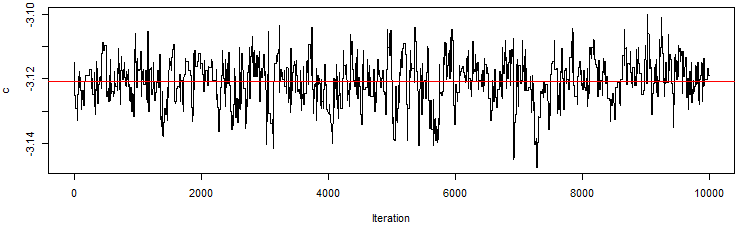


(b)

**Figure S2**. Monte Carlo Markov Chain sampling (after burn-in period) of $a$ and *c* in the situation with (a) *b* = 0.125 and (b) *b* = 0.225 from 1 October 2010 to 30 September 2017.

**3.2. Sensitivity analysis of parameter *L***

In the baseline, *L* is set to be 730(= 365$\times2$) days. In the sensitivity analysis, *L* is set to be 365 and 365 $\times3$ respectively. The inference of parameter *a* and *c* was calculated. The percentage reduction of influenza transmissibility during winter school breaks in Scenario 1 under low prevalence (95% *CI*) was estimated.

**Table S2**. Inference of parameter $a$ and *c* and percentage reduction in influenza transmissibility during winter school breaks with differing values of parameter *L*.

|  | *L* = 365 | *L* = 365$\times2$ (baseline) | *L* = 365$\times3$ |
| --- | --- | --- | --- |
| Parameter $a$ value  (95% *CI*) | 0.04078  (0.04068, 0.04089) | 0.03780  (0.03767, 0.03792) | 0.03575 (0.03561, 0.03593) |
| Parameter *c* value  (95% *CI*) | -2.53903  (-2.55420 ,-2.52527) | -3.10341  (-3.12622, -3.08390) | -3.40845  (-3.43055, -3.38152) |
| Percentage reduction of influenza transmissibility during winter school breaks in Scenario 1 under low prevalence (95% *CI*) | 3.9%  (0, 7.7%) | 5.0%  (1.9%, 7.8%) | 7.5%  (6.3%, 8.4%) |


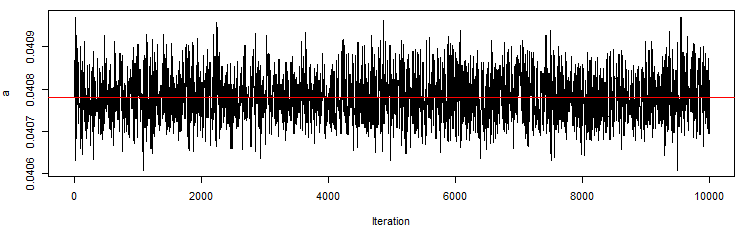


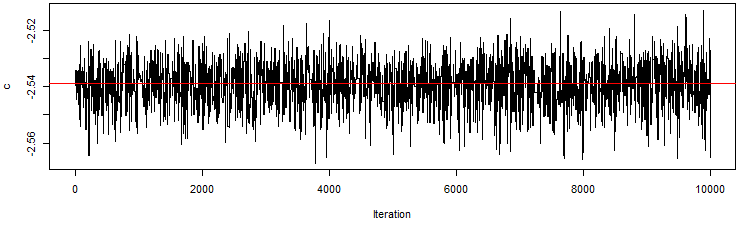


(a)


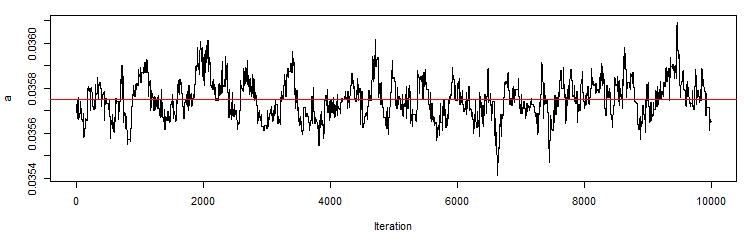


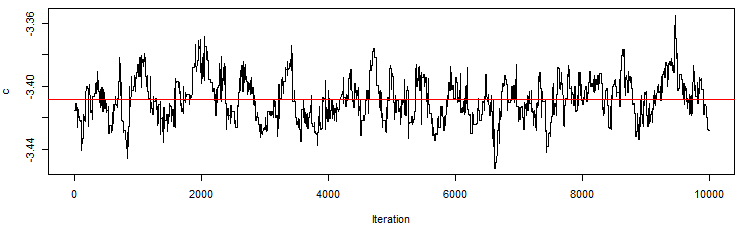


(b)

**Figure S3**. Monte Carlo Markov Chain sampling (after burn-in period) of $a$ and *c* in the situation with (a) *L =* 365; and (b) *L*$= 365 \times3$ from 1 October 2010 to 30 September 2017.

**3.3. Sensitivity analysis of parameter *D***

In the baseline, *D* is set to be 3 days. In the sensitivity analysis, *D* is set to be 2.5 and 3.5 respectively. The inference of parameter *a* and *c* was calculated. The percentage reduction of influenza transmissibility during winter school breaks in Scenario 1 under low prevalence (95% *CI*) was estimated.

**Table S3**. Inference of parameter $a$ and *c* and percentage reduction in influenza transmissibility during winter school breaks with differing values of parameter *D*.

|  | *D* = 2.5 | *D* = 3  (baseline) | *D* = 3.5 |
| --- | --- | --- | --- |
| Parameter $a$ value  (95% *CI*) | 0.03312  (0.03305,0.03320) | 0.03780  (0.03767, 0.03792) | 0.03230 (0.03218, 0.03243) |
| Parameter *c* value  (95% *CI*) | -6.08599  (-6.11743, -6.05109) | -3.10341  (-3.12622, -3.08390) | -3.33955  (-3.36164, -3.31516) |
| Percentage reduction of influenza transmissibility during winter school breaks in Scenario 1 under low prevalence (95% *CI*) | 10.1%  (8.2%, 12.3%) | 5.0%  (1.9%, 7.8%) | 7.2%  (5.8%, 8.2%) |

**
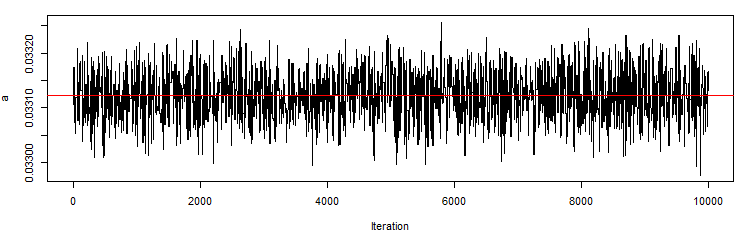
**

**
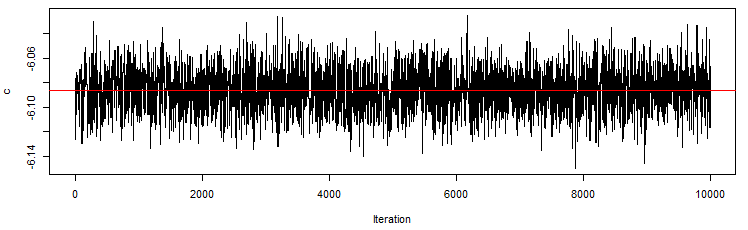
**

(a)


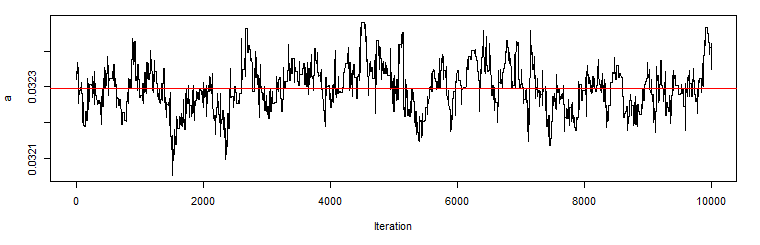


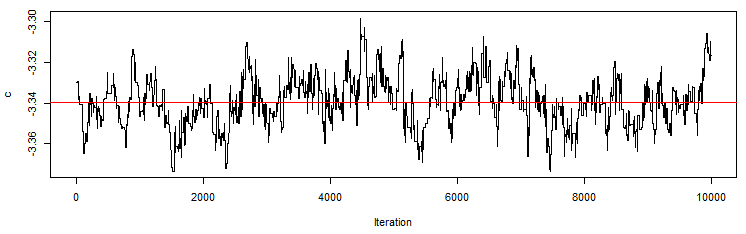


(b)

**Figure S4**. Monte Carlo Markov Chain sampling (after burn-in period) of $a$ and *c* in the situation with (a) *D =* 2.5; and (b) *D*$= 3.5$ from 1 October 2010 to 30 September 2017.

**3.4. Sensitivity analysis of parameter *w***

In the baseline, *w* is set to be 0.017 days, which means that the infection transmission efficiency parameter $\beta\left( t \right)=a$(1$+b$sin(*wt*+*c*)) is periodic function with period 1 year. In the sensitivity analysis, *w* is set to be 0.0157 and 0.021 respectively, which means that the infection transmission efficiency parameter $\beta\left( t \right)=a$(1$+b$sin(*wt*+*c*)) is periodic function with period 300 days and 400 days respectively. The inference of parameter *a* and *c* was calculated. The percentage reduction of influenza transmissibility during winter school breaks in Scenario 1 under low prevalence (95% *CI*) was estimated.

**Table S4**. Inference of parameter $a$ and *c* and percentage reduction in influenza transmissibility during winter school breaks with differing values of parameter *w*.

|  | *w* = 0.0157 | *w* = 0.017  (baseline) | *w* = 0.021 |
| --- | --- | --- | --- |
| Parameter $a$ value  (95% *CI)* | 0.03790 (0.03777, 0.03802) | 0.03780  (0.03767, 0.03792) | 0.03755  (0.03746, 0.03763) |
| Parameter *c* value  (95% *CI*) | -3.00971  (-3.03475, -2.98637) | -3.10341  (-3.12622, -3.08390) | -3.39067  (-3.40618, -3.37789) |
| Percentage reduction of influenza transmissibility during winter school breaks in Scenario 1 under low prevalence (95% *CI*) | 5.6%  (3.0%, 7.8%) | 5.0%  (1.9%, 7.8%) | 3.0%  (-2.1%, 7.6%) |

**
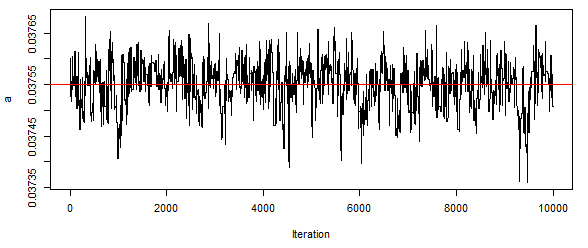
**

**
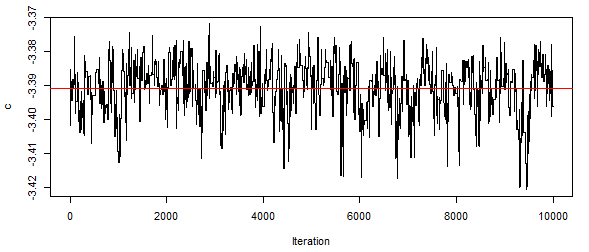
**

(a)


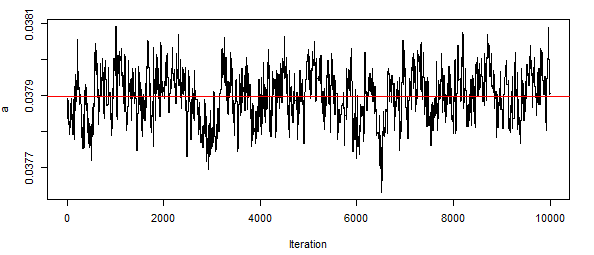


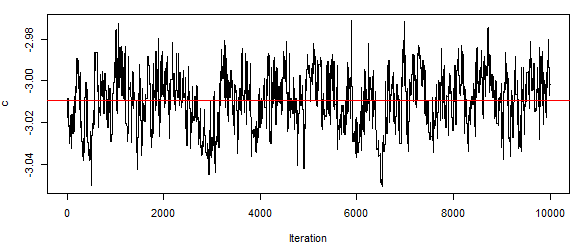


(b)

**Figure S5**. Monte Carlo Markov Chain sampling (after burn-in period) of $a$ and *c* in the situation with (a) *w =* 0.0157; and (b) *w =* 0.021 during the period from 1 October 2010 to 30 September 2017.

**References**

1. Zhang J, Litvinova M, Liang Y, et al. Changes in contact patterns shape the dynamics of the COVID-19 outbreak in China. Science 2020; 368: 1481-1486.
2. Jackson C, Mangtani P, Vynnycky E, et al. School closures and student contact patterns. Emerg Infect Dis 2011, 17(2): 245.
3. Cauchemez S, Valleron AJ, Boelle PY, et al. Estimating the impact of school closure on influenza transmission from Sentinel data. Nature 2008; 452: 750-754.
4. Birrell PJ, Ketsetzis G, Gay NJ, et al. Bayesian modeling to unmask and predict influenza A/H1N1pdm dynamics in London. Proc Natl Acad Sci USA 2011; 108:18238–43.
5. Bissiri PG, Holmes CC, Walker SG. A general framework for updating belief distributions. J R Stat Soc Series B Stat Methodol 2016; 78: 1103.
6. Olinky R, Huppert A, Stone L. Seasonal dynamics and thresholds governing recurrent epidemics. J. Math. Biol 2008; 56: 827-839.
